# Supplementary material for: Multi-omic latent variable data integration reveals multicellular structure pathways associated with resistance to tuberculin skin test (TST)/interferon gamma release assay (IGRA) conversion in Uganda
Source: BMC Genomics. 2025 Mar 18;26:265. doi: 10.1186/s12864-025-11407-1 (PMC11916984; doi:10.1186/s12864-025-11407-1)
Supplement: Supplementary file 1 — Supplementary Material 1 [file 12864_2025_11407_MOESM1_ESM.docx]

**Supplemental Figure Legends**

**Supplemental Figure 1. Availability of data types by patient.** A tile plot showing availability of each of the five integrated data modalities by patient. Only patients with all five data types (green) were used in the data integration methods presented.

**Supplemental Figure 2. Variance explained on each MOFA factor by dataset.** The variance explained by each dataset on each of all ten latent factors generated in MOFA. Bar colors represent the input dataset (ATAC-seq = coral, methylation = green, media-only RNA-seq = turquoise, Mtb-stimulated RNA-seq = blue, SNP = fuschia).

**Supplemental Figure 3. Sample-wise factor weights by group for all 10 MOFA factors.** Sample weights on all ten MOFA factors were tested for RSTR vs LTBI using a linear mixed effects model corrected for age, sex and genetic kinship. Black squares indicate groupwise means.

**Supplemental Figure 4. Top features by |weight| from datasets contributing > 5% of variance on Factors 1-3.** Groupwise differences in the five top weighted features on Factors 1-3 from datasets explaining > 5% of variance on these factors were tested using an ANOVA for RNA-seq, ATAC-seq, and methylation data and a Pearson’s chi-squared test for SNP data. Black squares indicate groupwise means.

**Supplemental Figure 5. Top five features by |weight| from datasets contributing > 5% of variance on Factor 4.** Groupwise differences in the five top weighted features on Factor 4 from datasets explaining > 5% of variance on this factor were tested using an ANOVA for RNA-seq, ATAC-seq, and methylation data and a Pearson’s chi-squared test for SNP data. Black squares indicate groupwise means.

**Supplemental Figure 6. Sample-wise variable values by group for all seven latent variables generated in non-MOFA integration methods.** Sample weights on all latent variables were tested for RSTR vs LTBI using a linear mixed effects model corrected for age, sex and genetic kinship. Black squares indicate groupwise means. P-value correction was performed within each of the three methods.

**Supplemental Figure 7. Enrichment result for reduced feature lists by factor.** Bars show counts of enriched gene sets with FDR < 0.2 by database. FDR values are calculated within database and factor.

**Supplemental Figure 8. Alluvial plot of GO terms from cross-database gene set clusters generated using the novel madRich method compared with rrvgo clustering of GO terms.** Colors indicate clusters assigned by madRich. Only GO terms can be clustered by rrvgo, so non-GO gene set terms used in generating madRich clusters are omitted.

**Supplemental Figures**

**
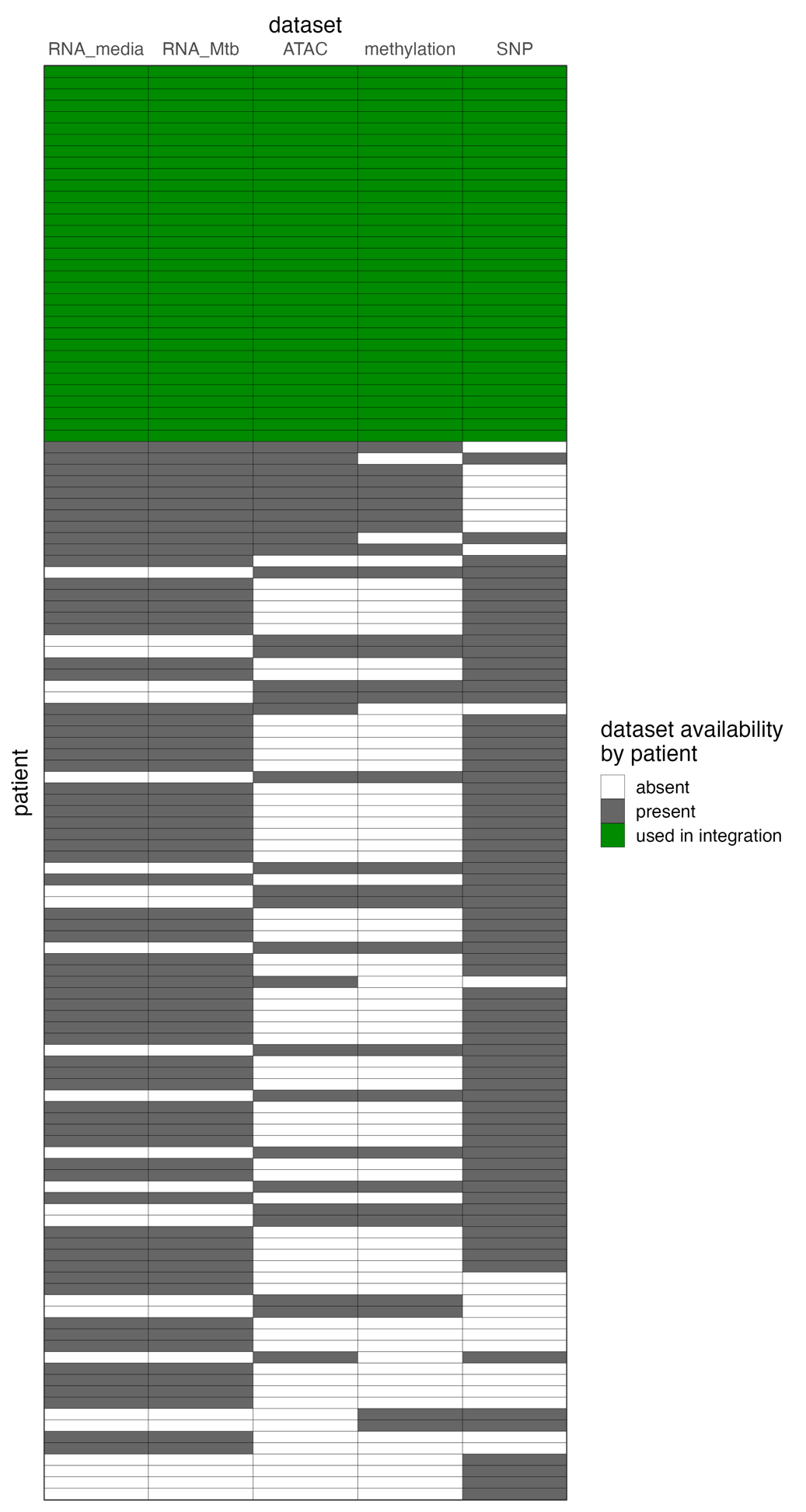
**

Supplemental Figure 1.


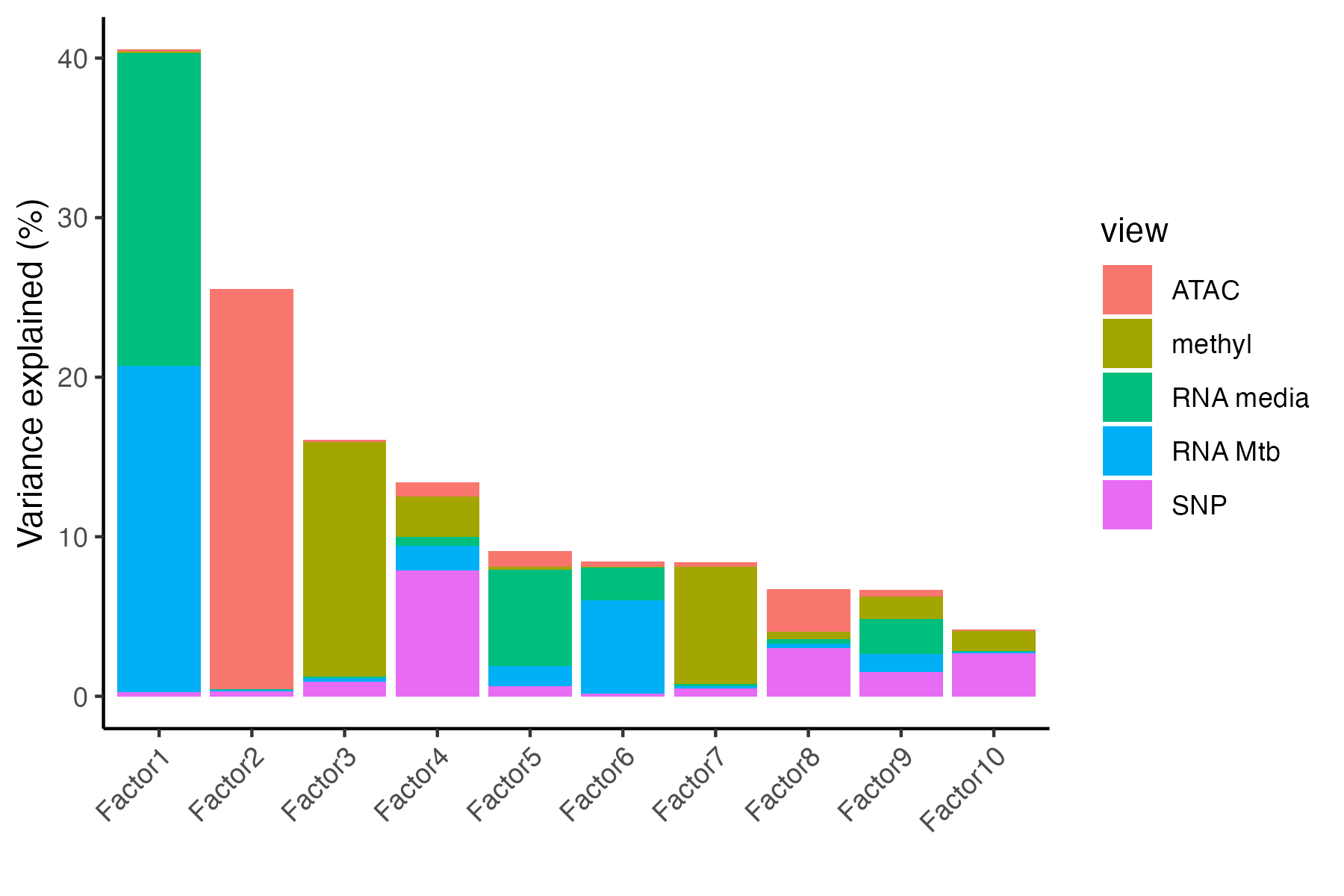


Supplemental Figure 2.


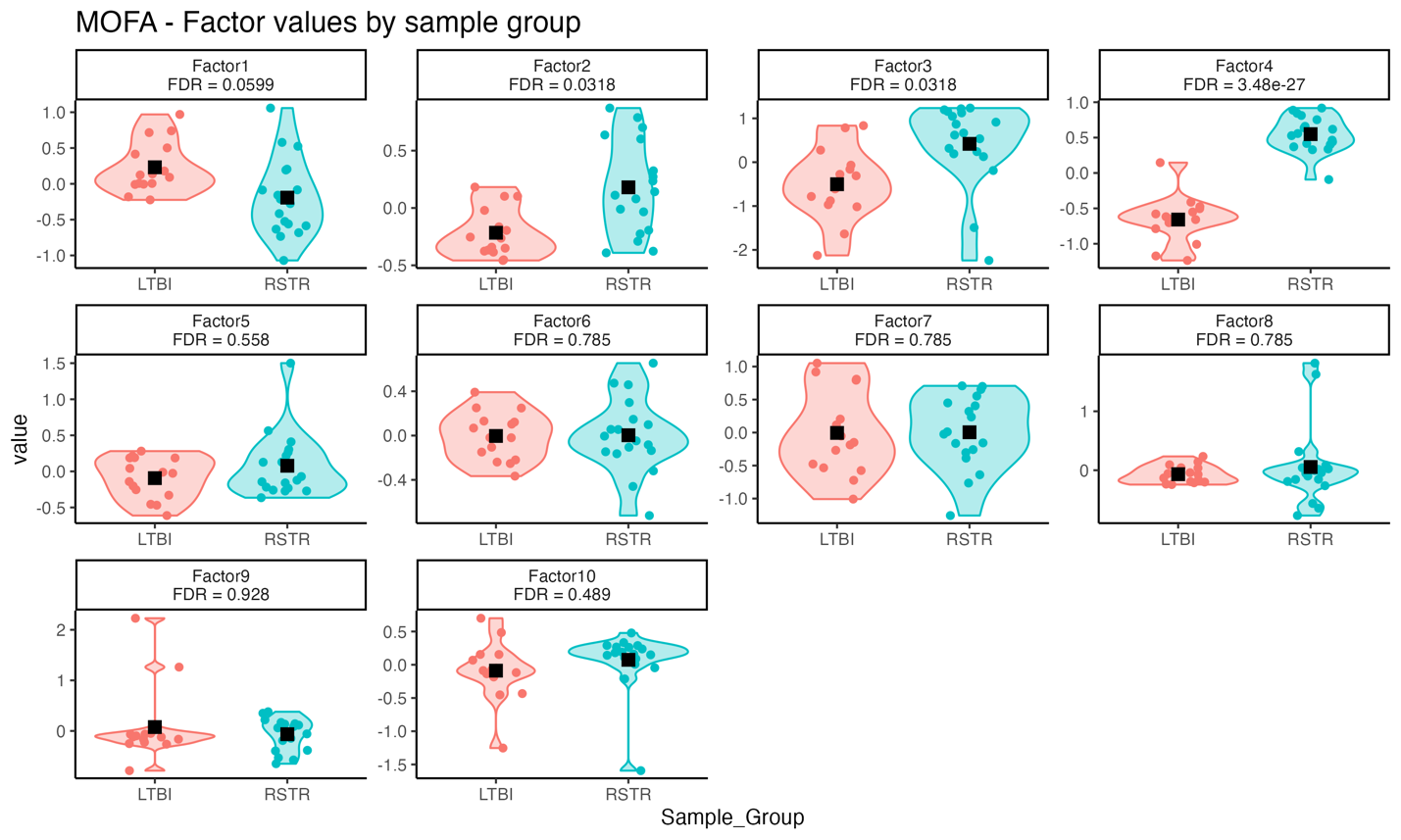


Supplemental Figure 3.


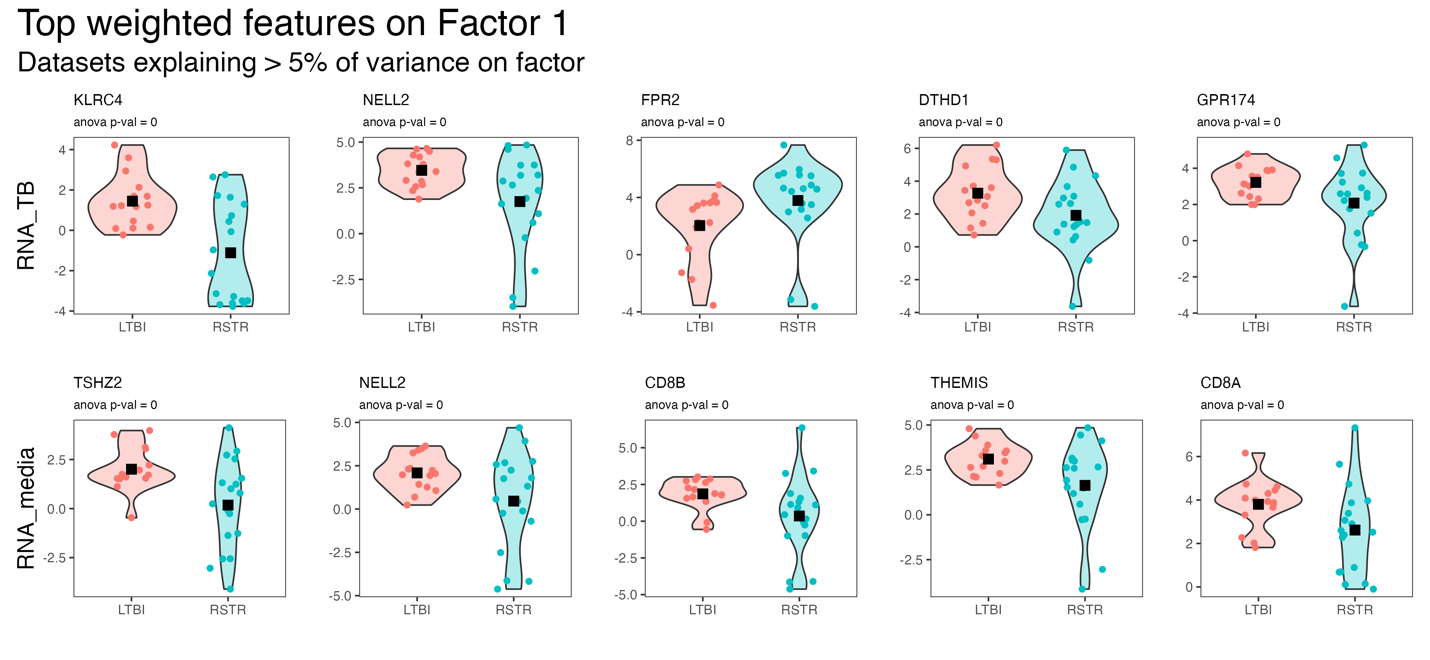


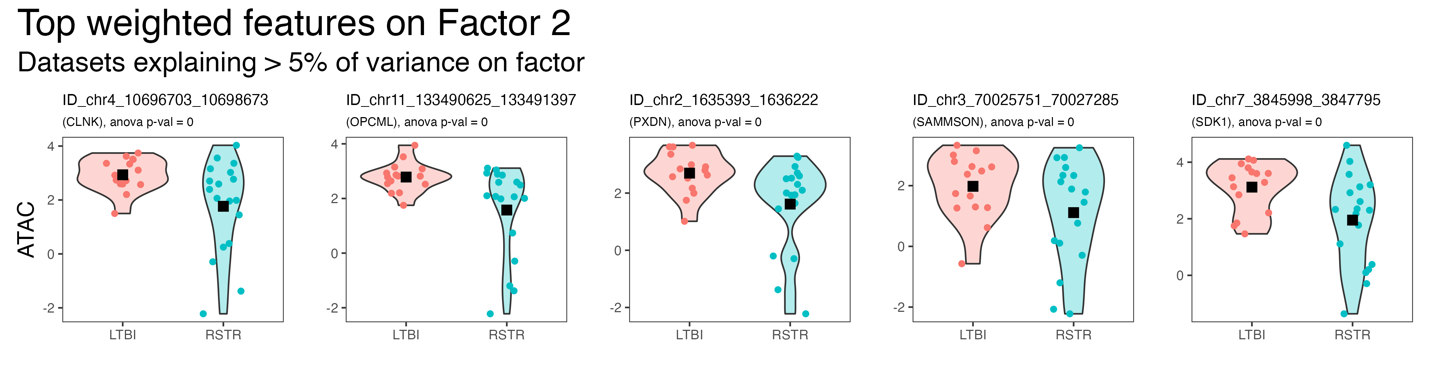


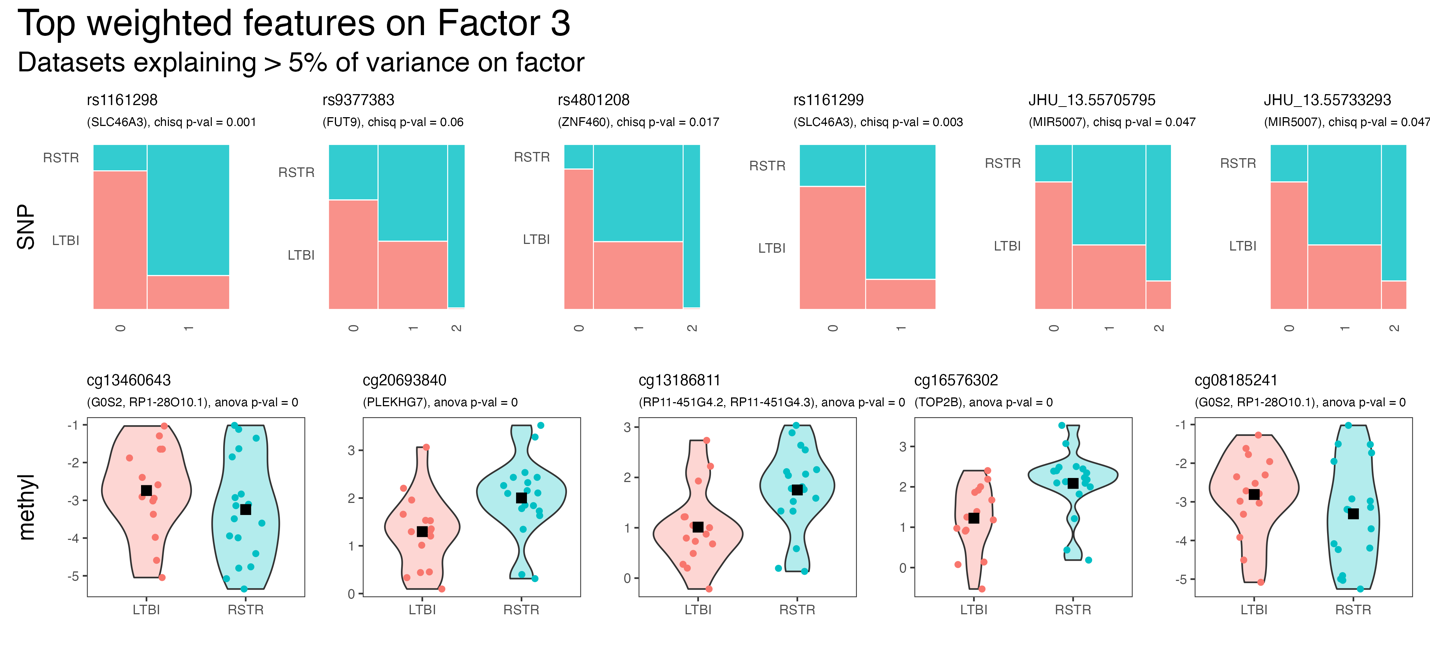


Supplemental Figure 4.


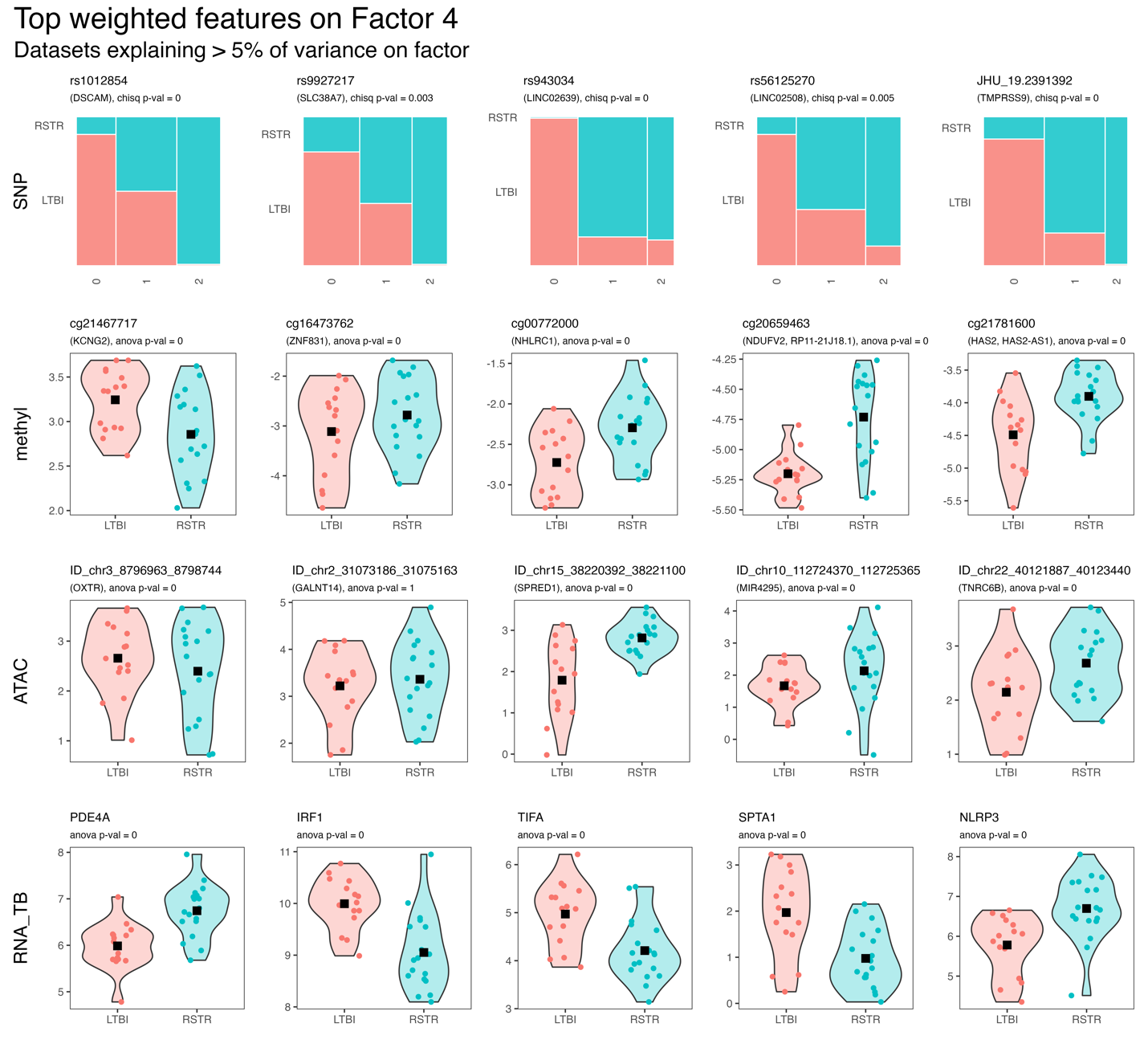


Supplemental Figure 5.


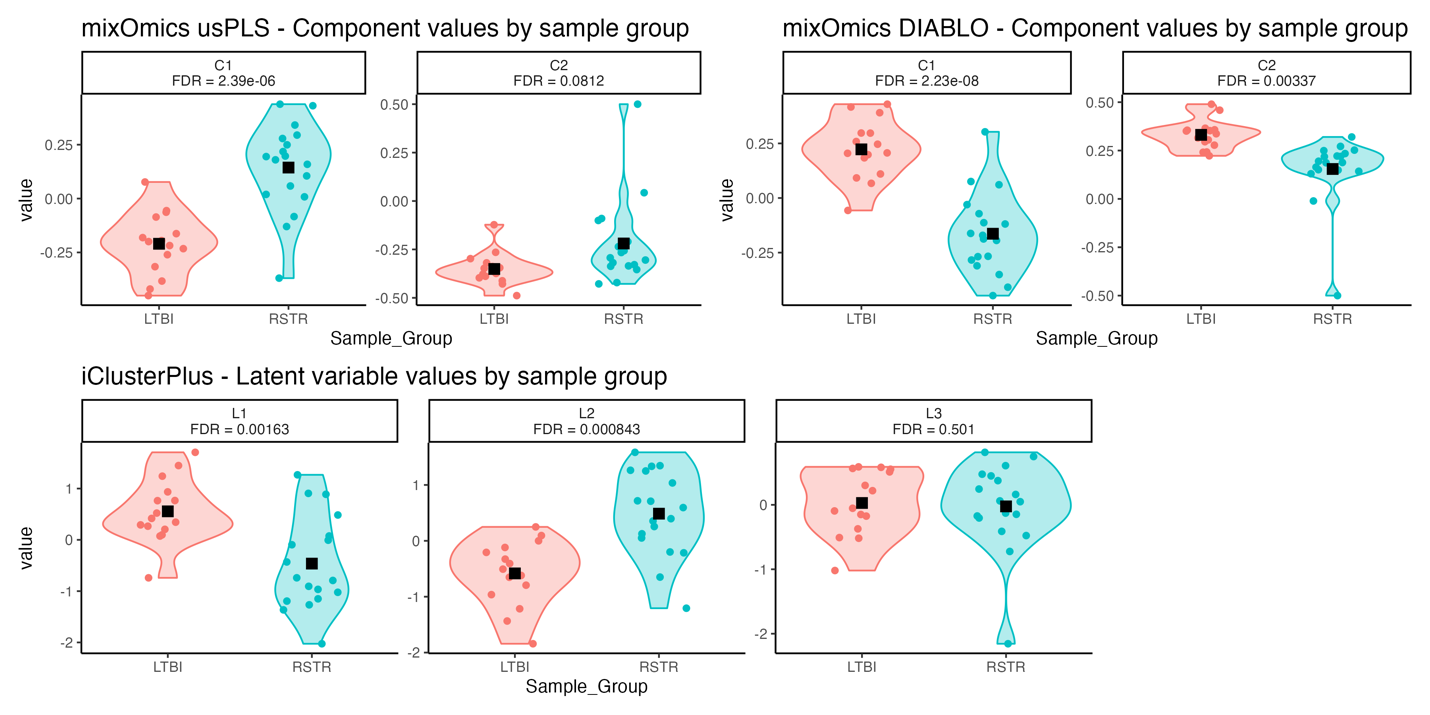


Supplemental Figure 6.


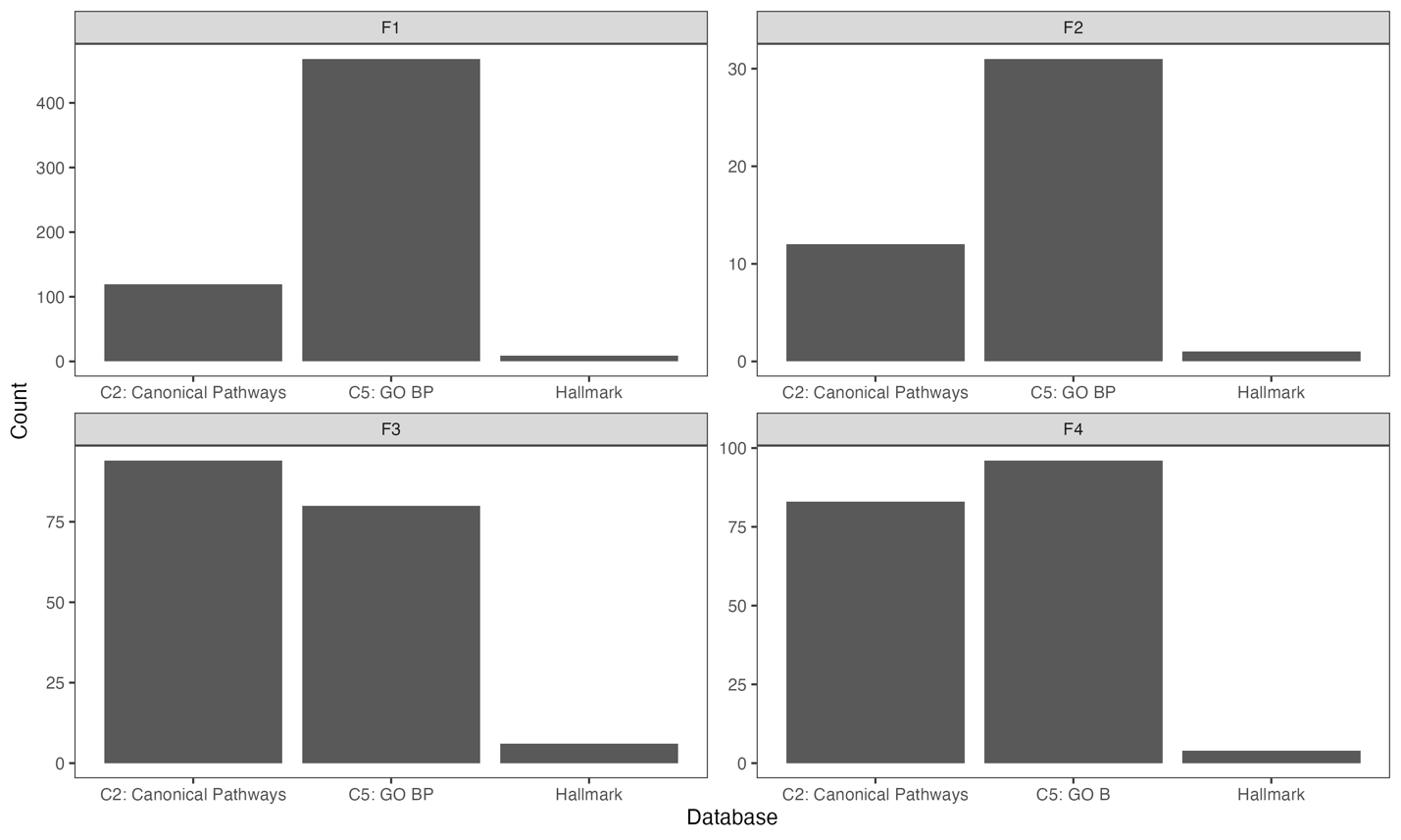


Supplemental Figure 7.


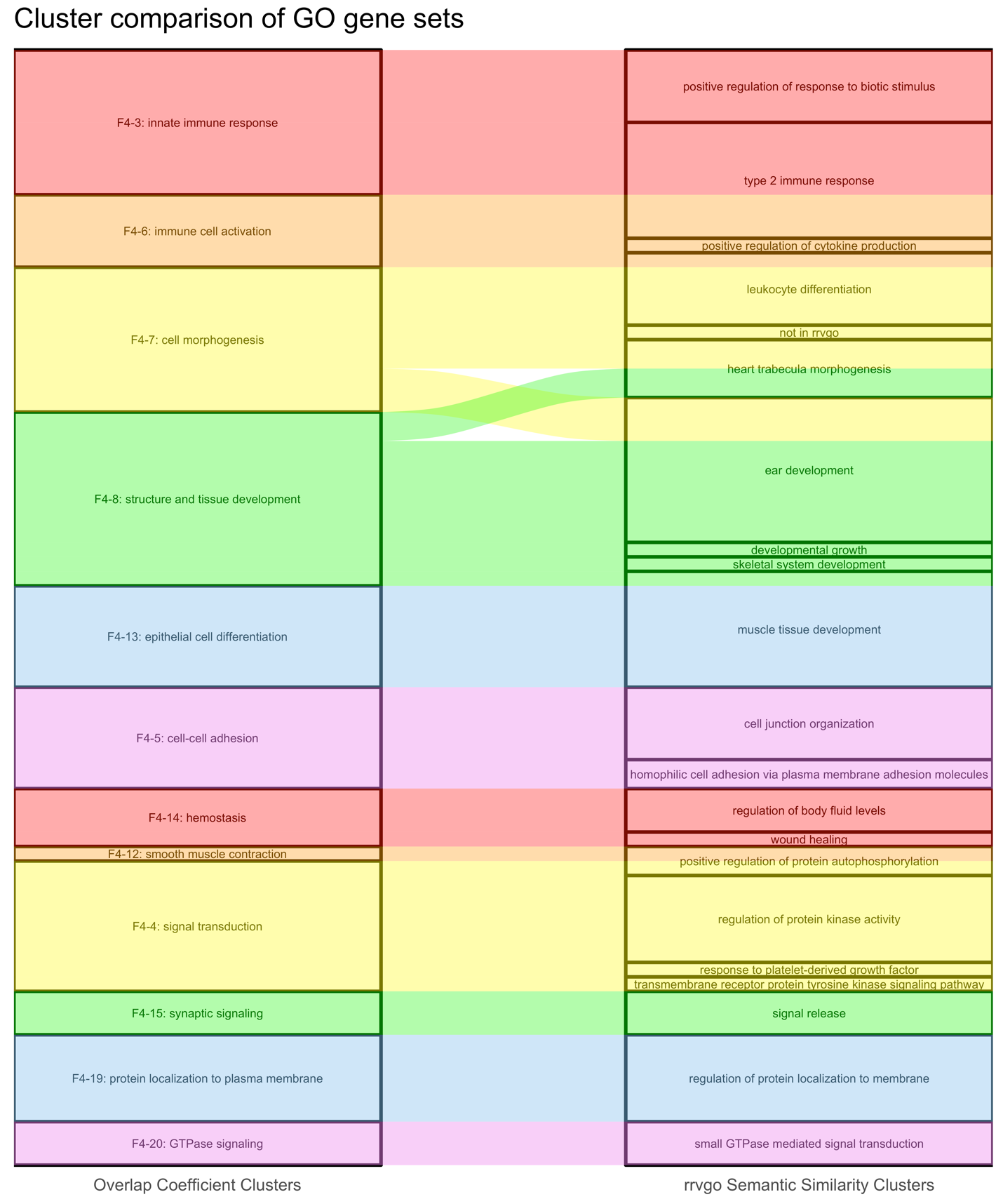


Supplemental Figure 8.

**Supplemental Tables**

**Supplemental Table 1. Latent variable statistics.**

| Method | Latent Variable | log FC | P value | FDR |
| --- | --- | --- | --- | --- |
| MOFA | Factor1 | -0.408 | 0.024 | 0.060^•^ |
|  | Factor2 | 0.341 | 0.010 | 0.032^*^ |
|  | Factor3 | 0.922 | 0.007 | 0.032^*^ |
|  | Factor4 | 1.179 | < 0.001 | < 0.001^***^ |
|  | Factor5 | 0.140 | 0.335 | 0.558 |
|  | Factor6 | -0.041 | 0.700 | 0.785 |
|  | Factor7 | -0.134 | 0.555 | 0.785 |
|  | Factor8 | 0.062 | 0.706 | 0.785 |
|  | Factor9 | -0.016 | 0.928 | 0.928 |
|  | Factor10 | 0.170 | 0.244 | 0.489 |
| mixOmics usPLS | C1 | 0.341 | < 0.001 | < 0.001^***^ |
|  | C2 | 0.113 | 0.081 | 0.081^•^ |
| mixOmics PLS-DA | C1 | -0.373 | < 0.001 | < 0.001^***^ |
|  | C2 | -0.164 | 0.003 | 0.003^**^ |
| iClusterPlus | L1 | -0.999 | 0.001 | 0.002^**^ |
|  | L2 | 0.903 | < 0.001 | 0.001^**^ |
|  | L3 | 0.149 | 0.501 | 0.501 |

**Supplemental Table 2. Summary of overlap in high-weight MOFA features and features with extreme values on latent variables generated with other methods.**

| **MOFA factor** | **Dataset (% variance explained on factor)** | **Number of MOFA features** | **Comparison feature source** | **Number of comparison features** | **Number of overlapping features**  **(% of MOFA features in overlap)** |
| --- | --- | --- | --- | --- | --- |
| Factor 1 | RNA Mtb (50%) | 177 | mixOmics DIABLO | 276 | 42 (23.7%) |
|  |  |  | mixOmics usPLS | 274 | 52 (29.4%) |
|  |  |  | iClusterPlus | 249 | 38 (21.5%) |
|  | RNA media (48%) | 169 | mixOmics DIABLO | 278 | 84 (49.7%) |
|  |  |  | mixOmics usPLS | 278 | 90 (53.3%) |
|  |  |  | iClusterPlus | 249 | 58 (34.3%) |
| Factor 2 | ATAC-seq (98%) | 605 | mixOmics DIABLO | 487 | 141 (23.3%) |
|  |  |  | mixOmics usPLS | 483 | 127 (21.0%) |
|  |  |  | iClusterPlus | 463 | 261 (43.1%) |
| Factor 3 | Methylation (91%) | 1198 | mixOmics DIABLO | 1046 | 475 (39.6%) |
|  |  |  | mixOmics usPLS | 1037 | 420 (35.1%) |
|  |  |  | iClusterPlus | 738 | 190 (15.9%) |
|  | SNP (6%) | 94 | mixOmics DIABLO | 1279 | 86 (91.5%) |
|  |  |  | mixOmics usPLS | 1291 | 77 (81.9%) |
|  |  |  | iClusterPlus | 1054 | 16 (17.0%) |
| Factor 4 | SNP (58%) | 973 | mixOmics DIABLO | 1279 | 445 (45.7%) |
|  |  |  | mixOmics usPLS | 1291 | 384 (39.5%) |
|  |  |  | iClusterPlus | 1054 | 153 (15.7%) |
|  | Methylation (19%) | 249 | mixOmics DIABLO | 1046 | 179 (71.9%) |
|  |  |  | mixOmics usPLS | 1037 | 125 (50.2%) |
|  |  |  | iClusterPlus | 738 | 16 (6.4%) |
|  | RNA Mtb (11%) | 40 | mixOmics DIABLO | 276 | 30 (75.0%) |
|  |  |  | mixOmics usPLS | 274 | 27 (67.5%) |
|  |  |  | iClusterPlus | 249 | 0 (0%) |
|  | ATAC-seq (6%) | 41 | mixOmics DIABLO | 487 | 31 (75.6%) |
|  |  |  | mixOmics usPLS | 483 | 13 (31.7%) |
|  |  |  | iClusterPlus | 463 | 1 (2.4%) |

**Supplemental Table 3. Summary of overlap in high-weight MOFA features and features with extreme values on latent variables generated with other methods.**

| **MOFA factor** | **Dataset (% variance explained on factor)** | **Number of MOFA features** | **Overlap** | **Number of features (% of features)** |
| --- | --- | --- | --- | --- |
| Factor 1 | RNA Mtb (50%) | 177 | At least one other method | 85 (48.0%) |
|  |  |  | At least two other methods | 41 (23.2%) |
|  |  |  | All three other methods | 6 (3.4%) |
|  | RNA media (48%) | 169 | At least one other method | 139 (82.2%) |
|  |  |  | At least two other methods | 84 (49.7%) |
|  |  |  | All three other methods | 9 (5.3%) |
| Factor 2 | ATAC-seq (98%) | 605 | At least one other method | 337 (55.7%) |
|  |  |  | At least two other methods | 132 (21.8%) |
|  |  |  | All three other methods | 60 (9.9%) |
| Factor 3 | Methylation (91%) | 1198 | At least one other method | 668 (55.8%) |
|  |  |  | At least two other methods | 372(31.1%) |
|  |  |  | All three other methods | 45 (3.8%) |
|  | SNP (6%) | 94 | At least one other method | 90 (95.7%) |
|  |  |  | At least two other methods | 77 (81.9%) |
|  |  |  | All three other methods | 12 (12.8%) |
| Factor 4 | SNP (58%) | 973 | At least one other method | 611 (62.8%) |
|  |  |  | At least two other methods | 334 (34.3%) |
|  |  |  | All three other methods | 37 (3.8%) |
|  | Methylation (19%) | 249 | At least one other method | 197 (79.1%) |
|  |  |  | At least two other methods | 114 (45.8% |
|  |  |  | All three other methods | 9 (3.6%) |
|  | RNA Mtb (11%) | 40 | At least one other method | 35 (87.5%) |
|  |  |  | At least two other methods | 22 (55.0%) |
|  |  |  | All three other methods | 0 (0%) |
|  | ATAC-seq (6%) | 41 | At least one other method | 31 (75.6%) |
|  |  |  | At least two other methods | 13 (31.7%) |
|  |  |  | All three other methods | 1 (2.4%) |

**Supplemental Table 5. Common features between reduced MOFA feature lists for Factors 1-4 and significant features from individual analyses of the integrated datasets.**

| **Factor** | **Comparison dataset** | **N features in comparison dataset** | **MOFA view** | **N features in reduced MOFA feature lists** | **N shared features** | **Shared features** | **HGNC symbols of shared features** |
| --- | --- | --- | --- | --- | --- | --- | --- |
| Factor 1 | RNA-seq | 260^A^ | RNA media | 84 | 0 |  |  |
|  | RNA-seq | 260^A^ | RNA Mtb | 41 | 4 | IFNG | IFNG |
|  |  |  |  |  |  | NLRP6 | NLRP6 |
| Factor 2 | ATAC-seq | 2^A^ | ATAC-seq | 132 | 0 |  |  |
| Factor 3 | Methylation | 324^B^ | Methylation | 372 | 0 |  |  |
|  | SNPs | 40^C^ | SNP | 77 | 0 |  |  |
| Factor 4 | SNPs | 40^C^ | SNP | 334 | 0 |  |  |
|  | Methylation | 324^B^ | Methylation | 114 | 5 | cg05713859 | AC006077.3, PCBD2 |
|  |  |  |  |  |  | cg06442199 | BRDT |
|  |  |  |  |  |  | cg11783376 | PCDHGA1, PCDHGA2, PCDHGA3, PCDHGA4, PCDHGA5, PCDHGA6, PCDHGA7, PCDHGA8, PCDHGB1, PCDHGB2, PCDHGB3, PCDHGB4, PCDHGB5 |
|  |  |  |  |  |  | cg07317843 | ABLIM1 |
|  |  |  |  |  |  | cg20109624 | PKD1L2 |
|  | RNA-seq | 261^A^ | RNA Mtb | 22 | 5 | FCAR | FCAR |
|  |  |  |  |  |  | IRF1 | IRF1 |
|  |  |  |  |  |  | IRF8 | IRF8 |
|  |  |  |  |  |  | MXD1 | MXD1 |
|  |  |  |  |  |  | SECTM1 | SECTM1 |
|  | ATAC-seq | 2^A^ | ATAC-seq | 13 | 0 |  |  |

^A^ RNA-seq & ATAC-seq FDR < 0.2

^B^ methylation FDR < 0.2 in either probe list or list of probes in differentially methylated regions

^C^ SNP *P* < 5e-5
